# *In vitro *characterization of cells derived from chordoma cell line U-CH1 following treatment with X-rays, heavy ions and chemotherapeutic drugs

**DOI:** 10.1186/1748-717X-6-116

**Published:** 2011-09-14

**Authors:** Takamitsu A Kato, Akihisa Tsuda, Mitsuru Uesaka, Akira Fujimori, Tadashi Kamada, Hirohiko Tsujii, Ryuichi Okayasu

**Affiliations:** 1 Research Center for Charged Particle Therapy, National Institute of Radiological Sciences, 4-9-1 Anagawa, Inage-ku, Chiba-shi, 263-8555 Japan; 2 International Open Laboratory, Particle Radiation Molecular Biology Unit, National Institute of Radiological Sciences, 4-9-1 Anagawa, Inage-ku, Chiba-shi, 263-8555 Japan; 3 Department of Environmental & Radiological Health Sciences, Colorado State University, Fort Collins, CO 80523 USA; 4 Nuclear Professional School, University of Tokyo, Tokyo, 113-8656 Japan

## Abstract

**Background:**

Chordoma, a rare cancer, is usually treated with surgery and/or radiation. However, very limited characterizations of chordoma cells are available due to a minimal availability (only two lines validated by now) and the extremely long doubling time. In order to overcome this situation, we successfully derived a cell line with a shorter doubling time from the first validated chordoma line U-CH1 and obtained invaluable cell biological data.

**Method:**

After isolating a subpopulation of U-CH1 cells with a short doubling time (U-CH1-N), cell growth, cell cycle distribution, DNA content, chromosome number, p53 status, and cell survival were examined after exposure to X-rays, heavy ions, camptothecin, mitomycin C, cisplatin and bleocin. These data were compared with those of HeLa (cervical cancer) and U87-MG (glioblastoma) cells.

**Results:**

The cell doubling times for HeLa, U87-MG and U-CH1-N were approximately 18 h, 24 h and 3 days respectively. Heavy ion irradiation resulted in more efficient cell killing than x-rays in all three cell lines. Relative biological effectiveness (RBE) at 10% survival for U-CH1-N was about 2.45 for 70 keV/μm carbon and 3.86 for 200 keV/μm iron ions. Of the four chemicals, bleocin showed the most marked cytotoxic effect on U-CH1-N.

**Conclusion:**

Our data provide the first comprehensive cellular characterization using cells of chordoma origin and furnish the biological basis for successful clinical results of chordoma treatment by heavy ions.

## Background

Chordoma is a rare malignant bone tumor accounting for only 1 to 4% of all primary malignant bone tumors [1]. Chordoma originates from notochordal remnants and has slower local growth and metastasizes less frequently than other bone and soft tissue malignant tumors [2]. Chordoma is not easy to control because of its anatomic location and propensity for spreading extensively. Complete radical resection produces better local control compared with subtotal resection and chemotherapy [1,2]. Some case studies reported that photon, proton, and charged particle carbon radiotherapy may delay possible recurrence after incomplete resection and may also be able to control the tumor [3-13]. A phase II study of 9-nitro-camptothecin in patients with advanced chordoma showed that it possessed modest activity in delaying progression with unresectable or metastatic chordoma [14]. Several reports suggested that PI3K/AKT/TSC1/TSC2/mTOR pathway and EGFR are potential therapeutic targets for chordoma [15,16]. One report showed that the combination with topoisomerase II inhibitor razoxane enhances the effectiveness of chordoma radiotherapy [17].

It is sometimes difficult to perform complete radical resection of chordoma tumors, depending on anatomic location or grade of tumor spreading. Because of the lower effectiveness of chemotherapy, radiotherapy is a useful treatment tool, and thus information on cellular radiosensitivities to photon and/or charged particles is urgently needed.

Despite the accumulation of data from the clinical side, there is a scarcity of information from the biology side because of the difficulty in obtaining basic cell biological data from the two currently available chordoma lines; the first cell line has been available for the last few years and the second one became available from the Chordoma foundation a few month ago. Another big obstacle is extremely long doubling time of chordoma cells. The first validated chordoma cell line, U-CH1, isolated by a German group, presented a long cell doubling time (~ 7 days) and chromosome instability and rearrangement [18]. U-CH1-N, a subpopulation derived from U-CH1 chordoma cells at National Institute of Radiological Sciences (NIRS), has acceptably shorter cell doubling time that enabled us to carry out *in vitro *cell biological research such as clonogenic cell survival assay. This study is the first to report the measurement of *in vitro *cellular radiosensitivity, heavy ion biological effectiveness, and responses to chemotherapy agents for a sacral chordoma cell line.

## Methods

### Cell lines and culture conditions

The chordoma cell line U-CH1 was kindly supplied by the Chordoma Foundation in Greensboro, NC, USA. U87-MG and HeLa cell lines were obtained from ATCC, USA. Cells were cultured in MEM-alpha (Gibco, Japan) supplemented with 10% fetal bovine serum (FBS, Sigma, Japan) and 1% antibiotics and antimicotics (Gibco, Japan), and they were maintained at 37°C in a humidified atmosphere of 5% CO_2 _in air.

### U-CH1-N cells and cell doubling time

Original U-CH1 cells had 7 days of doubling time in Iscove/RPMI (4:1) medium with 10% FBS in collagen-coated flasks [18]. In order to perform clonogenic colony formation assay, at least 7 cell divisions are required to obtain colony containing more than 50 cells. If we use the original U-CH1, it will take at least 2 months to get countable colonies. Therefore, we adapted U-CH1 in alpha-MEM medium supplemented with 10% FBS under normal culture conditions in tissue culture plastic flasks, similar to the other two cell lines. After three weeks we isolated fast growing subpopulation of U-CH1, and designated as "U-CH1-N" (N for NIRS). To measure the cell doubling time, cells were seeded at 5000 cells per T12.5 flask, and their number was counted at regular intervals.

### Comparison of parental and subpopulation of U-CH1, chromosome and p53 analysis

U-CH1-N cells were verified for their characteristics on karyotyping compared with their original U-CH1 cells. U-CH1-N cells were cultured with 0.1 μg/ml Colcemid for 6 hours to harvest metaphase chromosomes. Samples were treated in hypotonic solution, 75 mM KCl, for 20 min at 37°C and fixed in 3:1 (methanol: acetic acid) fixation solution three times. Spread metaphase chromosomes were stained with Giemsa solution, and the chromosome number was observed under a microscope.

Genomic DNA from parental U-CH1 and faster growing subpopulation U-CH1-N was isolated with Qiagen Blood & Cell Culture DNA mini kit (Qiagen, Japan). The genomic regions of the p53 gene were amplified by PCR using KOD plus polymerase (TOYOBO, Japan) with the following primers: hTP53AF (5'-ccattcttttcctgctccacaggaagccga-3') and hTP53BR (5'-ggctaagctatgatgttccttagattaggt-3') for exons 2 - 9, hTP53CF (5'-ctgtataggtacttgaagtgcagtttctac -3') and hTP53CR (5'-ttgtaaactaacccttaactgcaagaacat -3') for exons 10 and 11. The conditions of PCR were: 94°C, 2 min, 35 cycles of 94°C (15 sec) and 68°C (4 min). The amplified DNA fragments (approximate 3.6 kb and 1.5 kb) were subjected to sequencing reaction using the p53 exon-specific primers supplied by Nippon Gene (Toyama, Japan) and Big-Dye Terminator Cycle Sequencing FS Ready Reaction Kit V3.1 (Applied Biosystems, Foster City, CA). The nucleotide sequence was evaluated by genetic analyzer PRISM 310 (Applied Biosystems) and verified on both strands. The nucleotide sequence data of TP53 determined in the present study were deposited to DDBJ/EMBL/Genbank as a following accession ID; AB511810.

### Flow cytometry

Randomly dividing sample cultures were fixed in 70% ethanol and kept at -20°C until analysis. PI-stained 10,000 cells were analyzed by BD FACSCalibur (Beckton Dickinson, Japan) to obtain the DNA content histogram. Cell cycle characteristics were analyzed by Modfit program on Mac OS 9. The DNA content was compared with Chinese Hamster Ovary cells that have diploid DNA content and were calculated as 50 as an arbitrary unit.

### Irradiation and chemical treatment for Colony formation assay

Cells were irradiated with TITAN x-ray irradiator with 200 kVp, 20 mA, 0.5 cm of Al and Cu filter (Shimadzu, Japan). Heavy ion treatment was performed by HIMAC (Heavy Ion Medical Accelerator in Chiba). The accelerated ions used in this study were carbon ions (290 MeV/n), neon (400 MeV/n), silicon (490 MeV/n), argon (500 MeV/n), and iron ions (500 MeV/n). The details concerning the beam characteristics of carbon-ion beams, biological irradiation procedures, and dosimetry have been described elsewhere [19,20]. We used several kinds of beams having different LET values, using Lucite absorbers with various thicknesses to change the energy of the beams. At the sample position, we estimated the LET values of carbon (13, 30, 50, 70 keV/μm), neon (31, 70, 120 keV/μm), silicon (55, 150, 250 keV/μm), argon (100 keV/μm), and iron (200 keV/μm). Taking fragmentations into consideration, dose was calculated from fluence [21-23]. Asynchronously dividing cells cultured in T12.5 flasks were irradiated at room temperature. For chemical treatment, cycling cells in T12.5 culture flasks were exposed to series of concentration of bleocin, a single component of bleomycin family group A (Calbiochem, Japan), which induces DNA strand breaks, camptothecin (CPT, Sigma, Japan) which is a Topoisomerase I inhibitor, mitomycin C (MMC, Funakoshi, Japan) which induces DNA crosslink, or cisplatin (Nippon Kayaku, Japan) which induces DNA crosslink for 1 hour at 37°C.

After exposure to ionizing radiation or chemical treatment, cells were trypsinized and re-plated in P-100 cell culture dishes. HeLa and U87-MG cells were cultured for 10 to 14 days, and U-CH1-N cells were kept in an incubator for 3 to 4 weeks. Plating efficiency of U-CH1-N, U87-MG, and HeLa cells were 4.8%, 32%, and 70%, respectively. After colonies were formed, cells were fixed with 100% ethanol and stained with crystal violet solution. Colonies were observed under microscope and colonies containing more than 50 cells were counted as survivors. Cell survival assay was carried out 2 to 4 times independently. Radiation exposed cell survival curves were fitted with linear quadratic model by PRISM5 software on MacOSX10.6. Error bars indicate standard error of the means.

## Results

### Cellular doubling time, chromosome number, and p53 status of U-CH1-N

**Figure 1:**
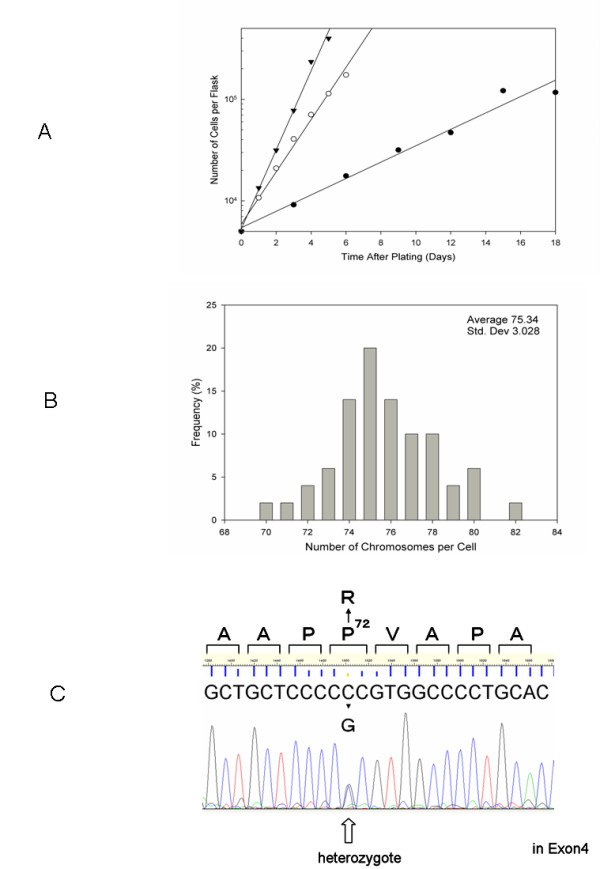
**Characters of cell lines**. A) Growth curves for three cell lines. Black reverse triangle indicates HeLa cells, white circle indicates U87-MG glioma cells, and black circle presents chordoma origin U-CH1-N cells. B) Chromosome number of chordoma origin U-CH1-N cells. 50 metaphase chromosomes were scored to obtain average chromosome number. C) The substitution C at 412 (NM_000546) to 'G.' was detected in the 4th exon of the p53 gene in U-CH1-N cells. Note that 'C' from wild-type allele is also detected. Deduced amino acid sequence is indicated at the top, where the mutation deduces Proline at 72 to Arginine.

The original U-CH1 cell line had a 7-day doubling time under culture medium and conditions originally used. We used the identical cell culture conditions for all three different tumor cell lines to avoid complexities arising from different growth conditions among them. The doubling time for U-CH1-N derived from U-CH1 at NIRS was about three days as against 7 days for the parental U-CH1 cell line. This reduced doubling time is still significantly longer than 21.5 hours for U87-MG and 18 hours for HeLa cells (Figure 1A). This shortened doubling time for U-CH1-N enabled us to carry out essential in vitro experiments including the colony formation assay to determine cell survival fraction against ionizing radiation and anti-tumor chemicals.

Our chromosome analysis of U-CH1-N cells showed that the distribution of chromosome numbers are practically identical to the numbers measured for the original U-CH1 cells (Figure 1B) [18]. Original U-CH1 had 75 chromosomes per cell on average, and our U-CH1-N cells averaged 75.34 chromosomes per cell.

The DNA sequencing data of the p53 gene of U-CH1-N was compared with the human wild-type TP53 gene MM_000546. It was revealed that one allele of p53 had a mutation carrying a C > G substitution at nucleotide residue 412 within exon 4, converting the corresponding amino acid from proline to arginine (Figure 1C). Parental U-CH1 carried exactly the same heterozygous mutation in p53 gene.

### Cell Cycle Distribution and DNA Content

Both cell cycle distribution and DNA profile were measured by a flow cytometer. The results are summarized in Table 1. DNA profile showed that U-CH1-N and HeLa were near tetraploid (about 100 and 90, respectively) compared with almost normal diploid DNA content (about 60) of U87-MG. Cell cycle distribution in chordoma cells showed a significantly high ratio in G1-phase, very different from the DNA profile patterns of the other two cell lines. These showed a greater number of cells in G1-phase (75%) and a smaller number in S-phase (13.3%). The slow growth speed of U-CH1-N may have a relationship with the long resting time before DNA synthesis in G1-phase.

**Table 1:** Cell cycle distribution and DNA contents of the three cell lines

Cell line	G1-phase	S-phase	G2/M-phase	DNA content*
HeLa	52.10%	30.30%	17.60%	~90

U87-MG	64.70%	23.00%	12.30%	~60

U-CH1-N	75.00%	13.30%	11.70%	~100

Data were obtained by flow cytometry analysis. *Arbitrary unit, a standard DNA content in normal diploid CHO cells (2n, G1-phase) is equal to 50.

### Cellular Radiosensitivity and Relative Biological Effectiveness

**Figure 2:**
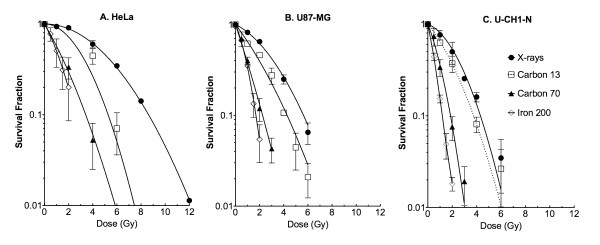
**Survival fraction after ionizing radiation exposure**. Cells were irradiated to X-rays or heavy ions having different LET. Black circle indicates X-rays, white square indicates Carbon LET 13 keV/μm, black triangle indicates Carbon LET 70 keV/μm, and white diamond indicates Iron LET 200 keV/μm. Error bars indicate standard errors of the mean of three independent experiments.

**Figure 3:**
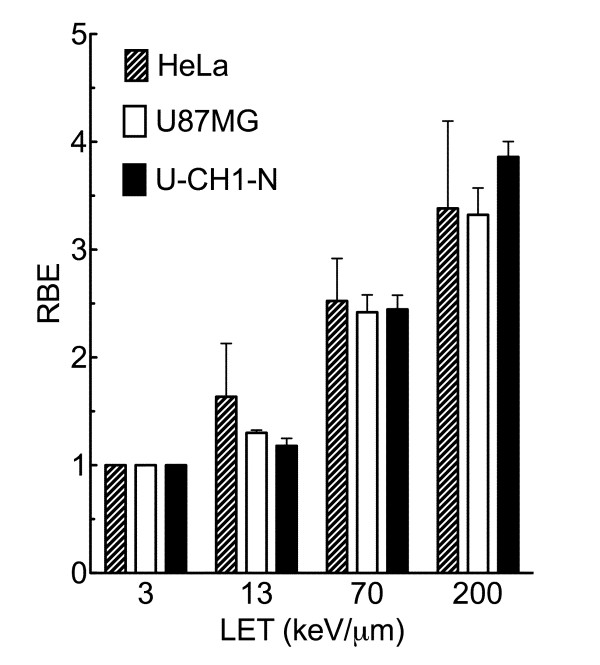
**LET and RBE curves for three cell lines**. RBE values were calculated from dose to get 10% survival fractions. LET 3 indicate 3 keV/μm for X-rays, 13 and 70 indicate 13 keV/μm or 70 keV/μm for carbon ions, and 200 indicates 200 keV/μm for iron-ions. Error bars indicate standard errors.

Asynchronous cell cultures were irradiated with various kinds of ionizing radiations (X-rays, carbon-ions 13 keV/μm, carbon-ions 70 keV/μm, iron-ions 200 keV/μm). Because of long cellular doubling time, the colony size of U-CH1-N was generally smaller than HeLa and U87 cells, even when a longer incubation time was allowed to form colonies. Nevertheless, by the time of fixation, we were able to observe U-CH1-N colonies containing more than 100 cells with or without irradiation. p53 mutated HeLa cells were the most resistant to all kinds of ionizing radiation among these cell lines; U87-MG and U-CH1 revealed similar radiosensitivity (Figure 2). From these D_10 _(radiation dose to kill 90% of irradiated cells) values, we calculated the relative biological effectiveness (RBE) of heavy charged particles compared to x-rays (Figure 3). RBE was obtained from D_10 _of x-rays divided by D_10 _of heavy ions with certain LET. RBE values of U-CH1-N cell line were not significantly different from ones of either HeLa or U87-MG by t-test.

### Extended Relative Biological Effectiveness Study for U-CH1-N

**Figure 4:**
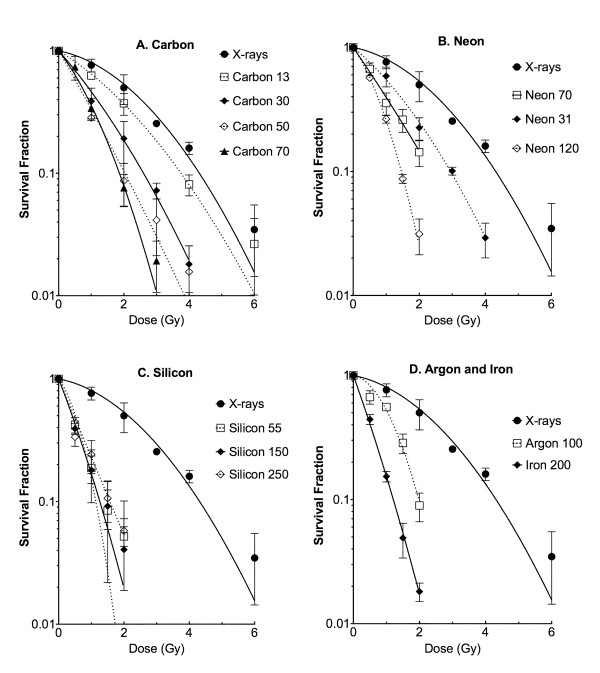
**Survival fraction of U-CH1-N cells after high LET heavy-ions**. A) X-rays and carbon-ions (LET 13, 30, 50, and 70 keV/μm), B) X-rays and neon-ions (LET 13, 70, and 120 keV/μm), C) X-rays and silicon-ions (LET 55, 150, and 250 keV/μm), and D) X-rays, argon-ions (LET 100 keV/μm), and iron-ions (LET 200 keV/μm). Error bars indicate standard error of the means from three or four independent experiments.

**Figure 5:**
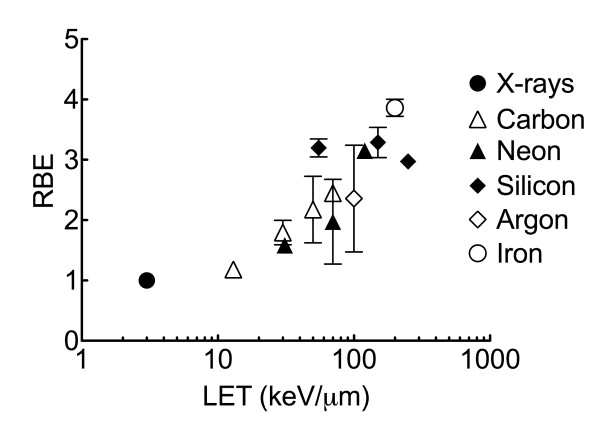
**Assembled LET and RBE relationship for U-CH1-N cells**. RBE values were calculated from 10% survival points. Error bars indicate standard error. Black circle indicates X-rays, white triangle; carbon-ions, black triangle; neon-ions, black diamond; silicon ions, white diamond; argon-ions, and white circle; iron-ions.

In order to understand the detailed RBE values in this chordoma cell line, 11 different qualities of photon and ion beams were employed to obtain cell survival curves (Figure 4). Calculated RBE values from D_10 _were plotted against LET (Figure 5). The RBE values increased up to LET near 200 keV/μm and decreased afterwards. The maximum RBE was approximately 3.86 at LET 200 keV/μm of iron beam.

### Sensitivity to Genotoxic Chemical Agents

**Figure 6:**
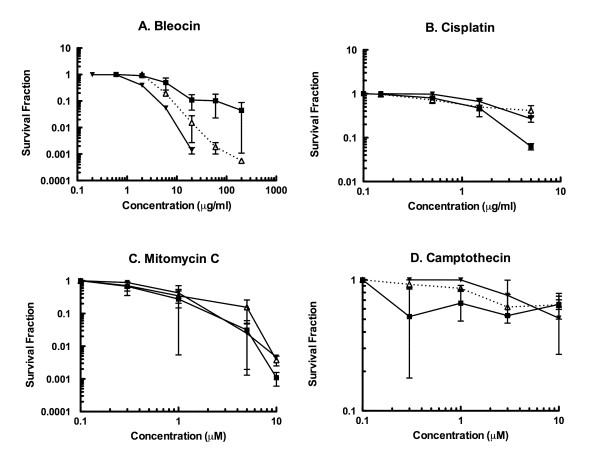
**Survival fraction after exposure to various genotoxic agents**. A) bleocin, B) cisplatin, C) mitomycin C, and D) camptothecin. Black square represents HeLa cells, white triangle indicates U87-MG cells, and black reverse triangle indicates U-CH1-N chordoma cell line. Cells were exposed to drugs at 37° for 1 hour. 2-4 independent experiments were carried out.

Figure 6 shows the survival curves of the four chemical agents. Although camptothecin, mitomycin C and cisplatin did not reveal strong cytotoxic effects for the particular cell lines under the treatment condition (1 hour, 37°C) we used, bleocin showed a distinct cell inactivation effect for U-CH1-N cells. This trend was also observed in U87-MG cells to less extent, but HeLa cells showed a very resistant phenotype to bleocin.

## Discussion

Chordoma is a rare tumor and information on its cellular radiobiology as well as chemotoxicity is still lacking. This study revealed that the chordoma cell line U-CH1-N *in vitro *cell culture condition was within the normal radiosensitivity range. We also examined the sensitivity to four different therapeutic agents. The higher sensitivity to ionizing radiation and bleocin, may suggest that chordoma cells are a good target for agents producing DNA double strand breaks. The results with other chemicals (cisplatin, mitomycin C, and camptothecin) indicate that chordoma cells are likely to have a normal repair mechanism other than the repair system needed for DNA double strand breaks. In general, p53 mutation confers a potential to change cellular radiosensitivity, increasing resistance due to reduced apoptosis induction by the inactivated p53 pathway [24-27]. HeLa cells have p53 mutation [28], while U87-MG cells have wild-type p53 [29] and show non-resistant phenotype. Judging from the cell survival data, we suspected that U-CH1-N cell line may have wild-type p53. We sequenced the p53 gene from parental U-CH1 and subpopulation U-CH1-N, and found that both cell lines retain a wild-type allele of p53 gene, although our sequence result exhibited a heterozygous mutation C > G, causing an amino acid substitution of proline 72 to arginine (Figure 1). Since the substitution has not been reported to confer any dominant negative effects of the gene [30], we estimated that this mutation hardly affect cellular radiosensitivity from cell cycle checkpoint or apoptosis induction [31].

U-CH1-N had slow growing and poor plating efficiency compared with other two tumor cells. In spite of these problems, we were still able to evaluate the radiosensitivity of U-CH1-N. The majority of colonies without irradiation contained more than 200 cells (more than 8 doublings), and the most of the colonies from irradiated cells contained more than 100 cells (more than 7 doublings) for U-CH1-N. Small colonies with 10-20 cells (less than 4 doublings) observed after irradiation were eliminated from survivors. If chordoma cells in general would have normal radiosensitivity as observed in U-CH1-N, the regular photon radiation therapy may control chordoma easily, although the location and the size could be a problem. However, the recurrence seems to be a big problem for chordoma patients after conventional radiotherapy [2,5].

It is possible that the poor tumor control associated with chordoma may be due to hypoxic effects and/or cancer stem cells which are resistant to ionizing radiation and chemical agents in *in vivo *tumor environment [32,33]. Chordoma tumors tend to be very large when they are diagnosed because of unnoticeable symptoms during the early stage. It is reasonable to consider that chordoma tumors contain a large fraction of hypoxic area. Recently, a PET (positron emission tomography) study revealed a substantial volume of chordoma is hypoxic [34]. Hypoxic regions within tumors are known to be radioresistant [35-37]. The clinical use of heavy charged particles with a spread out Bragg peak (SOBP) containing LET higher than 50 keV/μm could overcome the hypoxic tumor fraction [21]. In general, at low LET irradiation such as X-rays or gamma-rays, the Oxygen Enhancement Ratio (OER) is between 2.5 to 3. As the LET increases, the OER falls slowly until the LET exceeds about 60 keV/μm, after which the OER decreases rapidly and reaches unity by the time the LET reaches to about 200 keV/μm [38]. High LET exposure could overcome low oxygen concentrations which give radio-resistance in tumor populations, and thus this kind of radiation can control tumors with a better efficiency, but increasing LET means also high RBE to normal tissue [39]. Therefore, with high RBE for tumor control and the reduced OER, chordoma becomes a very attractive target for heavy charged particle therapy. The successful treatment of chordoma by carbon ions at our institute may be attributed to such characteristics even SOBP carbon ions are not as high RBE or low OER as monoenergetic high LET carbon beam [23,40].

## Conclusion

This study has comprehensively characterized the first validated chordoma cell line, U-CH1. Our next step will be to test more cell lines to verify our results; in vivo xenograft model with U-CH1-N should also be considered in the near future. Nonetheless, this is the first report presenting the extensive *in vitro *cellular studies including radiation and chemical cell survival/toxicity curves with the cell line originating from chordoma.

## Declaration of competing interests

The authors declare that they have no competing interests.

## Authors' contributions

TAK and AT performed most of the experiments and analyzed the data. MU helped in experimental design. AF performed p53 sequencing experiment in Figure 1 and helped prepare the manuscript. TK and HT provided help in experimental design and preparation of the manuscript. TAK and RO oversaw all the experiments and prepared the manuscript.
